# The Physicochemical Characteristics of Prosthetic Materials and Their Influence on Their Clinical Properties

**DOI:** 10.1007/s10337-017-3420-1

**Published:** 2017-10-28

**Authors:** Katarzyna Adamska, Beata Strzemiecka, Rafał Brożek, Ryszard Koczorowski, Adam Voelkel

**Affiliations:** 1 Institute of Chemical Technology and Engineering, Poznan University of Technology, Berdychowo 4, 60-965 Poznan, Poland; 2 Department of Geriatric Dentistry, Poznan University of Medical Sciences, Poznan, Poland

**Keywords:** Inverse gas chromatography, Prosthetic materials, Surface properties, Solubility parameter

## Abstract

The use of elastic materials favours degradation of their surface. The period of their clinical usefulness is then shortened, and their further utilisation in the oral cavity may have the reverse effect. The surface properties of such material as well as the influence of the humidity on their surface are very important as they determine the prosthetic materials behavior in the mouth. The surface of such material should be resistant to water. Inverse gas chromatography is an accurate, sensitive technique for studying surface properties. Thanks to using a unique equipment specially designed for IGC technique, Surface Energy Analyzer, it was possible to characterize the surface at 0 and 80% of humidity. Our results show that increased humidity does not affect surface properties of studied prosthetic materials. Their ability to dispersive and specific interactions change in very limited degree. IGC experiment was also applied for the estimation of Hansen solubility parameters that indicate ability of a material to dispersive, polar, and hydrogen-bonding interactions. Relation between the surface characteristics and practical use of soft lining materials with implications for their clinical usefulness is also discussed.

## Introduction

In dentistry, soft lining materials are used in patients in whom extremely difficult conditions of the basal seat area are found. Thanks to their flexibility properties, these materials make a cushioning, elastic layer of the mucous part of the denture, which is conducive to less traumatic transfer of occlusal forces onto the base. A removable denture lined with elastic material does not exert traumatic influence on the soft, rigid, and pressure-sensitive oral mucosa, is more comfortable and facilitates adaptation by the patient [1].

Soft liners can be classified as temporary, for short-term use and definitive to obtain permanent cushion-like effect. Long-term soft lining materials may also be divided according to the chemical structure into silicone elastomers and plasticized acrylates and due to the way of polymerization into room-temperature and heat-temperature polymerized. Lining made with use of high-temperature polymerized materials can be performed only by dental technician in prosthetic laboratory (indirect method). Lining made with use of room-temperature polymerized materials can be done in a dentist’s office by a dentist, as well (direct method of lining).

In the course of their utilisation, elastic materials are subject to a constant impact of the changeable and difficult conditions of the oral environment [2]. While being used, they become more porous and hence the number of places vulnerable to denture plaque and calculus accumulation increases which favours adhesion of the Candida fungi—the main pathogens responsible for the development of prosthetic stomatopathies [3, 4]. The roughness and microporosity of the surfaces of elastic materials increase the risk of infection in spite of frequent and regular hygiene practices applied by the patient. The observed phenomena of diffusion, elution, and leaching of compounds from the elastic materials may additionally worsen their surface properties. This process is accompanied by water absorption which continues until balance is achieved. Then, the materials become harder, and lose their elastic properties and the ability to reverse deformations. The coarse and rough surface may also affect the aesthetics of the prosthetic restoration, especially if the patient smokes and drinks large amounts of coffee and tea. Although these materials maintain the desired elastic properties for quite a long time, periodic replacement thereof is recommended for hygienic reasons [5].

The soft lining materials used in the study are successfully applied in dentistry due to their desired physical and chemical properties. Biological durability, low toxicity, the ability to be moulded easily to obtain a required shape, the lack of macroscopic changes in the surface features, shape preservation for a possibly long period of time, low glass transition temperature, and good thermal and chemical resistance make these materials difficult to be replaced in clinical practice.

Due to the strain resulting from the use in the oral cavity environment, changes in the micromorphology of their surface are, however, detected in images obtained with a scanning electron microscope. Surface degradation is observed as early as a few days after application, in the form of a network of longitudinally oriented grooves and cracks [1, 6]. The depth of the hollows appearing on the surface of the liner may range from 30 to 60 μm within 7 months, making elastic materials more susceptible to microbial colonisation [3, 7, 8].

Increased roughness of the elastic material surface may irritate the pressure-sensitive and often atrophic mucous membrane, and, at the same time, create optimal conditions for the colonisation and development of pathogenic microorganisms in the oral cavity environment. Changes of the surface features may lead to gradual spatial disintegration of the material and have a negative effect on the mechanical properties of the elastic materials, too [1, 9].

The group of chemical compounds called plasticizers (softeners) which includes phtalans, aliphatic esters, polyesters, cyclic ethers, phosphates, etc., is responsible for the elasticity of soft lining materials. The plasticizers decrease in the glass transition temperature thanks to which the hard materials become rubbery and elastic. The plasticizers are loosely connected with a network of polymeric chains and that is why they usually dissolve or leach into the oral cavity, which causes the material to become hard just like the denture base acrylate [10].

The compounds emitted from lining materials may have a negative, toxic influence on the tissues of the human organism. World literature pays particular attention to the fact that esters of phtalan acid may perform the role of xenoestrogens, i.e., industrially made hormonally active compounds. Due to the potentially toxic influence of plasticizers, European Scientific Committee on Toxicity, Ecotoxicity, and Environment (CSTEE) has limited the tolerable daily intake (TDI) of phtalans to 0.1–0.37 mg kg^−1^ day^−1^ [11]. In 1976, McCabe [12] established the safe daily dose of phtalans to be maximally 85 µg. Graham et al. [13] examined two soft lining materials and evaluated the amount of the emitted plasticizers to be 8–13 mg g^−1^ in the in vitro studies and 32–122 mg g^−1^ in the in vivo studies.

Due to the fact that the surface characteristics may exert influence on the clinical usefulness of these materials for the medical and dental practice, it seems justified to conduct studies into the physical and chemical properties of selected soft lining materials with the use of Inverse Gas Chromatography (IGC). IGC is a method, providing the data describing both surface and bulk properties of material. Retention parameters (retention time or retention volume) measured at the temperature higher than *T*_g_ (glass transition temperature) of the examined material (polymer or polymer blend), results from the sum of surface and bulk sorption. Below *T*_g_, the main process is the adsorption of probe molecules on the surface of the examined stationary phase. Surface properties of IGC examined materials are most often described by its ability to dispersive and specific interactions. The first one is expressed by using the dispersive component of the surface energy $\gamma_{\text{s}}^{\text{d}}$, while the second one with the help of *K*_A_ and *K*_D_, parameters.

Acid–base characteristic of the surface was performed on the basis of acid, *K*_A_, and basic, *K*_D_, parameters. Parameters *K*_A_ and *K*_D_ are related to the energy of specific interactions (*ΔG*_sp_) between the examined surface and the test compound [14]. They are determined by the dependence:

1 $$ \Delta G_{\text{sp}} = K_{\text{D}} \cdot {\text{AN}}^{*} + K_{\text{A}} \cdot {\text{DN,}} $$

where: $K_{\text{A}} ,K_{\text{D}}$—parameters determining the ability of the tested material surface to act, respectively, as the electron donor and acceptor [14], DN—the donor number of the polar test compound [15], and ${\text{AN}}^{*}$—modified acceptor number [16].

*K*_A_, parameters is obtained by dividing Eq. (1) by ${\text{AN}}^{*}$:

2 $$ \Delta G_{\text{sp}} /{\text{AN}}^{*} = \left( {{\text{DN}}/{\text{AN}}^{*} } \right){\kern 1pt} \, \cdot K_{\text{A}} + K_{\text{D}} , $$

and calculated from the slope of the straight line $\Delta G_{\text{sp}} /{\text{AN}}^{*}$ vs. $DN/AN^{*} .$

*K*_D_ parameter is obtained by dividing Eq. (1) by ${\text{DN}}$:

3 $$ \Delta G_{\text{sp}} /{\text{DN}} = \left( {{\text{AN}}^{*} /{\text{DN}}} \right)\, \cdot K_{\text{D}} + K_{\text{A}} , $$

what allows its calculation as the slope of the straight line $\Delta G_{\text{sp}} /{\text{DN}}$ vs. ${\text{AN}}^{*} /{\text{DN}}$.

Acidity and basicity of solid surfaces are determined most often by the ratio of *K*_A_ and *K*_D_ parameters:

4 $$ S_{\text{C}} = K_{\text{A}} /K_{\text{D}} . $$

In condensed phases (solutions and solid materials), strong interactions exist between molecules, resulting in considerable (negative) potential energy. This energy is called the *molar cohesive energy* (− *E*) [17–20], related to the molar volume, and is called *cohesive energy density c*:

5 $$ c = - E/V. $$

The square root of cohesive energy density is called *solubility parameter* $\delta$. This term proposed by Hildebrand for non-polar systems was related to the enthalpy of an evaporation $\Delta H_{\text{w}}$ of solvents, as a measure of their intermolecular forces:

6 $$ \delta = \sqrt c = \sqrt {E_{\text{coh}} /V} = \sqrt {\left( {\Delta H_{\text{w}} - RT} \right)/V} , $$

where $\delta$ is the solubility parameter, $E_{\text{coh}}$ is the cohesive energy, $V$ the molar volume of a solvent, $R$ the gas constant, and $T$ the temperature.

Solubility parameter data are useful in the description and interpretation of different phenomenon, occurring between materials like, e.g., miscibility, compatibility, or adsorption.

Inverse gas chromatography technique enables the estimation of solubility parameter based on the model of adsorption, described by Snyder and Karger [21–23]. According to such model, the molecule of test solute “*i*” is adsorbed onto the surface of solid adsorbent “*j*”. The energy of adsorption *ΔE* ^A^ for the respective test solutes can be calculated using the following expression:

7 $$ - \Delta E^{\text{A}} = V_{i} \left( {E_{ij} } \right) = V_{i} \left( {E_{ii} } \right)^{1/2} \left( {E_{jj} } \right)^{1/2} = V_{i} \left( {\delta_{i} \delta_{j} } \right), $$

where: *V*_i_—molar volume of test solute, *E*_*ii*_ and *E*_*jj*_—densities of the energy of cohesion of test solute “*i*” and adsorbent “*j*”, and *E*_*ij*_—the density of the energy of interaction.

So-called Hansen solubility parameter (HSP) [24] is an extension of the Hildebrand solubility parameter to polar and hydrogen-bonding systems, where the cohesive energy can be considered as a sum of contributions from dispersion ($E_{\text{d}}$), polar ($E_{\text{p}}$), and hydrogen-bonding ($E_{\text{h}}$) interactions

8 $$ - E_{\text{coh}} = - E_{\text{d}} - E_{\text{p}} - E_{\text{h}} . $$

The total solubility parameter ($\delta_{T}^{{}}$) is expressed as follows:

9 $$ \delta_{\text{T}}^{2} = \delta_{\text{d}}^{2} + \delta_{\text{p}}^{2} + \delta_{\text{h}}^{2} , $$

where: $\delta_{\text{d}}$, $\delta_{\text{p}}$, and $\delta_{\text{h}}$ denote dispersion, polar, and hydrogen-bonding contribution, respectively.

The energy of adsorption is related to the specific retention volume by the equation:

10 $$ \ln V_{\text{g}} = - \left( {{{\Delta E^{A} } \mathord{\left/ {\vphantom {{\Delta E^{A} } {RT}}} \right. \kern-0pt} {RT}}} \right) + {\text{const}} . $$

According to Hansen concept:

11 $$ - \Delta E_{i}^{\text{A}} = V_{i} \left[ {\left( {E_{ij} } \right)_{\text{d}} + \left( {E_{ij} } \right)_{\text{p}} + \left( {E_{ij} } \right)_{\text{h}} } \right] $$

or

12 $$ - \Delta E_{i}^{\text{A}} = V_{i} \left( {\delta_{\text{d}}^{i} \delta_{\text{d}}^{j} + \delta_{\text{p}}^{i} \delta_{\text{p}}^{j} + \delta_{\text{h}}^{i} \delta_{\text{h}}^{j} } \right), $$

where $\Delta E_{i}^{\text{A}}$ is adsorption energy for ith test solute. HSP of the examined material, i.e., $\delta_{{_{\text{d}} }}^{j}$, $\delta_{{_{\text{p}} }}^{j}$, $\delta_{\text{h}}^{j}$ can be calculated, using experimental retention $V_{\text{g}}$ data for selected test solutes and multiple linear regression.

It should be noted that the results of examination of many materials as, e.g., starch, synthetic (biodegradable) polymers, and various pharmaceutical might be influenced by the relative humidity of the environment, i.e., the content of water vapour in the carrier gas. This influence was examined and reviewed in several papers [25–28]. The water adsorbing on the material under examination might (in the case of hydrophilic materials) significantly influence the values of IGC-derived parameters.

IGC is useful technique to use in studying biomaterials as composites, ceramic biomaterials. This technique was applied, e.g., for the examination of dentine restorative materials and their interactions with tooth tissues [29–31]. However, up to now, there are no data concerning IGC characterization of biomaterials being prosthetic lining materials. The aim of this work was to characterize the series of prosthetic lining materials and correlation of IGC-derived parameters with functional properties.

## Experimental

### Materials

Series of soft lining materials were examined to establish their stability under different conditions (Table 1).

**Table 1:** Materials used in the study

Soft lining material	Supplier (or manufacturer)	Chemical composition								
		CAS	Abbr.	Chemical name	Molecular formula	Molecular weight (g/mol)	Density (g/cm^3^)	Glass transition temp. (°C)	Melting point (°C)	Degree of crystallinity
Molloplast B	Detax	9011-14-7	PMMA	Poly(methyl methacrylate)	(C_5_O_2_H_8_)_n_	Varies	1.18	105	160	N/A
		2530-85-0	MAOPTMS	$\gamma$-methacryloxypropyltrimethoxysilane	C_10_H_20_O_5_Si	248.35	1.045	N/A	< − 50	N/A
Villacryl soft	Vertex Dental	80-62-6	MMA	Methyl methacrylate	C_5_H_8_O_2_	100.117	0.9337	N/A	− 47.55	N/A
		97-90-5	EGDMA	Ethylene glycol dimethacrylate	C_10_H_14_O_4_	198.218	1.055	N/A	− 40	N/A
		77-90-7	ATBC	Acetyl tributylcitrate	C_20_H_32_O_8_^−2^	400.468	1.046	N/A	− 80	N/A
		9011-14-7	PMMA	Poly(methyl methacrylate)	(C_5_O_2_H_8_)_n_	Varies	1.18	105	160	N/A
Silagum comfort	DMG Hamburg			Vinylsilicone						
		80-62-6	Aerosil	Silicon dioxide	SiO_2_	60.083	0.9337	N/A	1703	N/A
Mollosil	Detax	63148-62-9	PDMS	Polydimethylsiloxane	(C_2_H_6_OSi)_n_	208.479	965 kg	N/A	N/A	N/A
		7440-06-4		Platin catalyst	Pt	195.084	21.45	N/A	1768.3	N/A

### Inverse Gas Chromatography

Inverse gas chromatography experiments were carried out using SEA (Surface Energy Analyzer produced by Surface Measurement System Ltd., UK) equipped with a flame ionization detector (FID). The studied materials were placed in the silanized column (300 mm length and 4 mm I.D.) as a strips (20 mm length and 1–2 mm width) in a quantity of 250 mg.

The surface characteristic—calculation of $\gamma_{\text{s}}^{\text{d}}$, *K*_A_, and *K*_D_, was carried at 37 °C of the column oven. This measurement was performed at 0 and 80% of the relative humidity (0 and 80 RH). Temperatures of the column for HSP determination were 30, 40, 50, 60, 70, and 80 °C. HSP measurement was performed at 0% of humidity. The column was conditioned at a given temperature by 2 h. For all IGC measurements, the temperature of the detector and injector was 150 °C. Dead time was determined using methane as inert gas. The carrier gas was helium (flow-rate 15 cm^3^/min). The following compounds were used as test solutes: hexane (anhydrous 95%, Sigma-Aldrich), heptane (99%, Sigma-Aldrich), octane (99%, Fluka), nonane (99%, Sigma-Aldrich), decane (98%, Fluka), dichloromethane (99.8%, Sigma-Aldrich), ethanol (99.8%, Avantor), ethyl acetate (99.8%, Sigma-Aldrich), 1,4-dioxane (99.8% Sigma-Aldrich), and acetonitrile (99.8% Sigma-Aldrich).

Test solutes were injected in the amount assuring the work in infinite dilution in chromatographic column. It should be noted that examination of materials in infinite dilution region under relative humidity regime is not the same as finite concentration of IGC.

The dispersive component $\gamma_{\text{s}}^{\text{d}}$ of the surface energy of a solid can be estimated by several methods; here, the method proposed by Schultz and Lavielle was used [32]. Acid–base characteristic of the surface was performed on the basis of acid, *K*_*A*_, and basic, *K*_*D*_, parameters calculated from Eqs. (2) and (3). HSP of the examined material, i.e., $\delta_{{_{\text{d}} }}^{j}$, $\delta_{{_{\text{p}} }}^{j}$, and $\delta_{\text{h}}^{j}$, were calculated, through determination of $\Delta E^{\text{A}}$ from temperature dependence of using experimental retention $V_{\text{g}}$ data (Eq. 10) for selected test solutes and further multiple linear regression (Eq. 12).

## Results and Discussion

Parameters describing surface properties of studied prosthetic materials are presented in Table 2. All studied materials are characterized by relatively high value of $\gamma_{\text{S}}^{\text{D}}$ which means that they have strong ability for dispersive interactions. Much higher value of $\gamma_{\text{S}}^{\text{D}}$ has Silagum Comfort that can be the effect of the presence of alkyl groups on the surface of silicone. Its ability to specific interactions is limited. All materials has surface that acts as donor of electron (higher value of *K*_D_ than *K*_A_ parameter). Values of *K*_A_ parameter are low what means that all materials exhibit low ability to act as electron acceptor (low acidic properties). This observation is due to the electron donor groups on the surface of all studied materials such as C=O, C=C, (CO)O. The differences between materials are small although statistically valid. The most active material is Silagum Comfort. However, Villacryl Soft exhibits highest ability to specific interactions. The value of the sum (*K*_A_ + *K*_D_) equal to 0.689 (at 0% RH) is highest for this material. The lowest ability to specific interaction was found for Mollosil—(*K*_A_ + *K*_D_) equal to 0.580 (at 0% RH). However, it should be noted that the differences between the ability specific interactions for all materials are low but statistically valid. The most important is the fact that all studied prosthetic materials did not change significantly surface properties in humidity 80% (small or no changes of the values of determined parameters). The least changes in the values of all estimated parameters are observed for Silagum Comfort that can be explained by the presence of alkyl groups on the surface that makes it hydrophobic and resistant to water. The relatively most active material (Villacryl Soft) changes its properties in most significant way. *K*_A_ parameter for villacryl increased from 0.096 to 0.119 while *K*_D_ from 0.494 to 0.677. It caused a significant change of its character to more basic (decrease of *K*_A_/*K*_D_ ratio). Villacryl Soft is the mixture of polyesters that probable causes its sensitivity to the water. The least changes in the values of all estimated parameters are observed for Silagum Comfort. This material seems to be most stable in changing conditions as its surface is most hydrophobic and it is composed of the relatively less sensitive to water compounds (only Aerosil can adsorb water).

**Table 2:** IGC parameters characterizing the surface properties of studied prosthetic materials

Material	$\gamma_{\text{S}}^{\text{D}}$ [mJ/m^2^]	*K*_A_	*K*_D_	*K*_A_/*K*_D_
0 RH				
Molloplast B	61.3 ± 6.5	0.100 ± 0.007	0.464 ± 0.042	0.214 ± 0.007
Villacryl soft	53.6 ± 0.1	0.096 ± 0.001	0.494 ± 0.004	0.195 ± 0.001
Silagum comfort	91.5 ± 1.8	0.102 ± 0.001	0.418 ± 0.001	0.244 ± 0.003
Mollosil	67.1 ± 3.6	0.084 ± 0.002	0.308 ± 0.025	0.272 ± 0.015
80 RH				
Molloplast B	54.0 ± 2.0	0.093 ± 0.004	0.442 ± 0.038	0.210 ± 0.005
Villacryl soft	56.2 ± 2.1	0.119 ± 0.003	0.677 ± 0.005	0.176 ± 0.002
Silagum comfort	93.0 ± 3.0	0.102 ± 0.002	0.424 ± 0.003	0.241 ± 0.001
Mollosil	70.3 ± 1.4	0.084 ± 0.002	0.328 ± 0.006	0.256 ± 0.002

The significant changes in the surface properties of soft lining materials might indicate the tendency to degradation of their clinical properties.

The change in the surface features of elastic materials caused by an increase in the number of details may result from intensified water absorption [1, 33, 34]. In the study, increased absorption of all the examined materials has been observed; however, Villacryl Soft, classified as a plasticised acrylate, was characterized by the higher values of parameters describing their ability to specific interactions in comparison with silicone elastomers. The phenomenon of water absorption is intensified by the chemical compounds, including plasticisers, released from the acrylic materials into the oral environment. The exchange proceeds until balance is achieved, and in the first week, water absorption may amount to 0.2–5.6 mg/cm^2^, whereas solubility may be 0.03–0.4 mg/cm^2^, depending on the investigated material [35]. The results of the study may suggest that these materials can facilitate adhesion of microorganisms and chemical substances taken when meals are consumed, and the strength of the bond between the material and the denture base may weaken in the course of the use thereof [36].

On the basis of the information provided by the manufacturers, the presence of ethylene glycol dimethacrylate particles in Villacryl Soft can change flexible properties. This compound is added both to silicone and acrylate materials to improve the cross-linking ability of the polymeric chains. The increase in the number of the cross bonds causes a change of the mechanical properties. The glass transition temperature of the material rises, which makes the material harder, more fragile, and less elastic (i.e., elastic modulus increases).

The elastic properties of soft lining materials may also be affected by their polymerization method. Many authors claim that Molloplast B of which the polymerization proceeding at high temperature in a dental technique laboratory ensures a higher degree of polymerization in comparison to materials polymerizing at room temperature. The polymerization of such materials is then more complete [33, 37, 38].

Specific retention volume data of test solutes obtained for all temperatures were used for the calculation of the energy of adsorption and then Hansen solubility parameter of examined materials, according to Snyder–Karger model. Dispersive, polar, hydrogen-bonding components and total solubility parameter value of examined materials are presented in Table 3.

**Table 3:** Components and total solubility parameters values for examined materials

Material	HSP [MPa]^1/2^			
	$\delta_{\text{d}}$	$\delta_{\text{p}}$	$\delta_{\text{h}}$	$\delta_{\text{T}}$
Molloplast B	16.62 ± 0.15	17.21 ± 0.12	6.85 ± 0.17	24.87 ± 0.16
Villacryl soft	14.37 ± 0.10	14.63 ± 0.25	11.79 ± 0.22	23.65 ± 0.20
Silagum comfort	18.25 ± 0.08	18.81 ± 0.19	8.81 ± 0.30	27.65 ± 0.24
Mollosil	16.31 ± 0.09	18.48 ± 0.10	7.30 ± 0.14	25.71 ± 0.11

It can be seen that all examined materials have different ability to intermolecular interactions. For polydimethylsiloxane group of materials—Molloplast B and Mollosil, we can observe similar values for components and total solubility parameter. These two materials has also similar value of $\gamma_{\text{S}}^{\text{D}}$. Also for these materials, the lowest values of the hydrogen-bonding component are observed. The highest ability to dispersion interaction—18.25 [MPa]^1/2^ has Silagum. This may be related to the presence of the vinyl group, that increases ability to the dispersion and polar interaction of such material, which confirms the higher dispersion and polar component value—18.25 and 18.81 [MPa]^1/2^, respectively.

The highest total solubility parameter—27.65 [MPa]^1/2^ is observed for Silagum Comfort. Villacryl, representative of acrylic materials, shows the lower dispersion and polar HSP value, but it has the higher ability to interact through hydrogen bonding, as evidenced by the high values of $\delta_{\text{h}}$ from all examined materials. It is also characterized by the highest value of *K*_D_ parameter.

For Silagum Soft, the largest capacity for dispersion interactions confirms the highest values of dispersion component of solubility parameter $\delta_{\text{d}}$, but also $\gamma_{\text{S}}^{\text{D}}$ data. Similarly, for Villacryl Soft, showing the least capacity for this type of interaction, calculated $\delta_{\text{d}}$ and $\gamma_{\text{S}}^{\text{D}}$ values for IGC experiments are the lowest. The values of the solubility parameter can be used to estimate the magnitude of interactions between materials/compounds, e.g., their mutual solubility or miscibility. The similar values of the parameter indicate stronger interaction.

The HSP values for dental materials can be compared with water HSP, but it is hard to state exactly which data for water are correct—see Table 1.3 in Ref [24] and Table 7 in Ref [39], which includes solubility parameter for water, obtained using different procedures. HSP values for water differ depending on the analysis of their determination. Quoting Hansen—“The behavior of water often depends on its local environment, which makes general prediction very difficult”. HSP values for water differ depending on the analysis of their determination. The largest differences are observed for the hydrogen-bonding interactions—values calculated using SPHERE program are similar, while calculated from the energy of vaporization are significantly higher. The values of the total solubility parameter determined by the computer program are almost similar. HSP values for dental materials and water indicate that the energy of the dispersive interaction is similar, while the largest differences are observed for the hydrogen-bonding interactions. These values are much higher for water molecules. Although the total values of solubility parameters ($\delta_{\text{T}}$), the dental values, are more comparable (except data 1 in Table 4) to the values for water, these relations suggest the relative resistance of dental materials in water environment.

**Table 4:** Components and total solubility parameters values for water

	HSP [MPa]^1/2^				Ref.
	$\delta_{\text{d}}$	$\delta_{\text{p}}$	$\delta_{\text{h}}$	$\delta_{\text{T}}$	
Data 1	15.5	16.0	42.3	47.8	[24]
Data 2	15.1	20.4	16.5	30.3	[24]
Data 3	18.1	17.1	16.9	30.1	[24]
Data 4	19.5	17.8	17.6	31.7	[39]

Conclusions based on concerning the activity of soft lining materials based on HSPs results are in agreement with those reported earlier for surface parameters (Table 2). One should note that *K*_A_ and *K*_D_ represent surface ability to act as electron acceptor and donor, respectively, while HSPs $\delta_{\text{p}}$ and $\delta_{\text{h}}$ expressed the tendency to polar and hydrogen-bonding interactions.

The study gave different results of both surface parameters and Hansen Solubility Parameters, although these materials are classified into the same group in terms of their chemical composition. The diversity may be caused by different ways of preparing each material as well as mixing the components of the preparations. Molloplast B, which polymerises at high temperatures, is characterized by greater cross-linking of residual monomers; therefore, its surface is harder and more resistant to the influence of the external environment [40].

The qualitative differences in the molecular composition, varying values of the surface energy and hydrophilicity may cause the investigated materials to be characterized by different biological properties. Nakamoto et al. have confirmed that the higher the surface energy, the greater the ability of microorganisms to adhere to the elastic liner surface [41].

## Conclusions

IGC proved to be an appropriate and useful technique for studying surface properties of the prosthetic materials and the stability of their surface under the influence of humidity. On the basis of IGC data obtained at different values of humidity, Silagum should be the most stable material taking into account the water influence. It also possesses the highest total solubility parameter value and potential for to non-polar and permanent dipole–permanent dipole interactions. The other tested prosthetic materials were characterized by the surface more sensitive to water, but the changes of the values of the IGC parameters under the influence of water were not large. Obtained Hansen solubility parameter data indicate greater potential for tested materials to the dispersive and polar intermolecular interactions.

The solubility parameter is a thermodynamic description of the system and can, therefore, be used in assessing the impact of various conditions on the behavior of the tested materials and explanations of intermolecular phenomena. It is currently thought that bacterial adhesion is started by electrostatic attraction, hydrophobic reactions, and the Van der Waals forces between the bacterial cell membrane and the surface of the elastic material [42, 43]. This process, being the initial adhesion, is unspecific and reversible. Numerous studies present the correlation between the build-up of bacterial plaque and hydrophobicity or hydrophilicity of the surface of lining materials, surface free energy, or the specific properties of the bacteria and the carrier’s organism [44]. The studies by Quirynen et al. conducted in vivo have proven that hydrophobic surfaces such as Teflon accumulate ten times fewer bacteria than the hydrophilic enamel surfaces [45]. Quirynen et al. and Absolom et al. believe that most of the bacteria found in the oral cavity demonstrate high free energy, so are more willing to settle on hydrophilic surfaces [45, 46]. The experiments have revealed that the highest value of dispersive component of surface energy $\gamma_{S}^{D}$ equal to 91.2 [mJ/m^2^] has been obtained for Silagum Comfort, which confirms strong hydrophobic properties of this material. The lowest values $\gamma_{S}^{D}$ equal to 53.6 [mJ/m^2^] have been obtained for Villacryl Soft. The high hydrophilicity of this material may deepen the mechanical surface irregularities (roughness) and impair the ability to deform elastically and flow (texture and rheological properties), which may, in turn, encourage the adhesion of pathogenic microorganisms, including fungi from the *Candida* genus, to the surface of the elastic material [47]. The hydrophilicity, high value of free energy, and wettability of the material surface may favour the build-up of bacterial plaque on its surface [45].

The examined lignin materials seem to be relatively stable in water environment. These are characterized by relatively strong ability to dispersive interactions (high value of $\gamma_{\text{S}}^{\text{D}} )$. All materials have surface acting as donor of electron. Examined materials did not change significantly their surface properties in humidity equal to 80%. Hansen solubility parameter data indicate greater potential of tested materials to the dispersive and the polar intermolecular interactions. HSPs results are in agreement with those found for surface parameters. Surface properties and Hansen solubility parameter of soft lining materials can be correlated with the specific properties of the bacteria and adhesion of pathogenic microorganisms to the surface.
